# Unraveling the Serotonergic Mechanism of Stress-Related Anxiety: Focus on Co-Treatment with Resveratrol and Selective Serotonin Reuptake Inhibitors

**DOI:** 10.3390/biomedicines12112455

**Published:** 2024-10-25

**Authors:** Vadim E. Tseilikman, Olga B. Tseilikman, Marina N. Karpenko, Dmitrii S. Traktirov, Daria A. Obukhova, Vladislav A. Shatilov, Maxim S. Zhukov, Gennady V. Manuilov, Oleg N. Yegorov, Maxim R. Aristov, Ilya A. Lipatov, Irina A. Buksha, Alexandr E. Epitashvili, Anton A. Pashkov, Jurica Novak

**Affiliations:** 1 Higher Medical and Biological School, South Ural State University, 454080 Chelyabinsk, Russia; 2 Faculty of Fundamental Medicine, Chelyabinsk State University, 454001 Chelyabinsk, Russia; 3 Zelman Institute of Medicine and Psychology, Novosibirsk State University, 630090 Novosibirsk, Russia; 4 Pavlov Department of Physiology, Institute of Experimental Medicine, 197376 Saint Petersburg, Russia; 5 Federal Neurosurgical Center, 630048 Novosibirsk, Russia; 6 Department of Data Collection and Processing Systems, Novosibirsk State Technical University, 630048 Novosibirsk, Russia; 7 Centre for Informatics and Computing, Ruđer Bošković Institute, 10000 Zagreb, Croatia

**Keywords:** chronic stress, resveratrol, noradrenaline, serotonin, dopamine, hippocampus, receptors, mRNA

## Abstract

**Background/Objectives:** In post-traumatic stress disorder (PTSD), anxiety-like symptoms are often associated with elevated noradrenaline levels and decreased serotonin. Selective serotonin reuptake inhibitors (SSRIs) are frequently used to treat anxiety, but elevated serotonin has been observed in some anxiety disorders. This study investigates stress-induced anxiety as an immediate effect of chronic stress exposure using the predator stress paradigm. **Methods:** We examined serotonin levels, serotonin transporter (SERT), and 5-HT_3*A*_ receptor gene expression in response to stress. The effects of SSRIs (paroxetine, sertraline) and resveratrol on these parameters were also analyzed, alongside co-treatment with resveratrol and sertraline. **Results:** Chronic stress exposure led to a significant increase in serotonin levels and upregulation of SERT and 5-HT_3*A*_ receptor expression. SSRIs failed to prevent anxiety or reduce serotonin levels, partly due to suppressed SERT expression. Resveratrol downregulated SERT and 5-HT_3*A*_ expression less than SSRIs but effectively reduced anxiety and restored serotonin, likely by upregulating MAO-A expression. Co-treatment with resveratrol and sertraline produced the strongest anxiolytic effect. **Conclusions:** Elevated serotonin and increased expression of SERT and 5-HT_3*A*_ receptor genes are key factors in stress-related anxiety. Resveratrol and SSRIs target these mechanisms, suggesting potential therapeutic strategies for anxiety disorders. Future research will focus on further elucidating the serotonergic mechanisms involved and identifying new anxiolytic drug targets.

## 1. Introduction

Anxiety is an emotion that plays a crucial role in behavioral adaptation in everyday life. Intense anxiety, characterized by the “fight or flight” response, promotes survival in the face of danger. However, when excessive, anxiety can become pathological. Anxiety disorders are the most common psychiatric illnesses, leading to profound disability, significant suffering, and a reduced quality of life [1]. Stress is a key risk factor for the development of anxiety disorders, with stress-related anxiety affecting serotonin levels in different regions of the brain [2].

Serotonin is a ubiquitous neurotransmitter responsible for regulating various aspects of mood through its interaction with serotonin receptors (5-HT receptors) [3]. There are seven classes of 5-HT receptors: 5-${\mathrm{HT}}_{1}$ to 5-${\mathrm{HT}}_{7}$. All except 5-${\mathrm{HT}}_{3}$ are G-protein-coupled receptors (GPCRs) located on the cell surface. Serotonin binding to these receptors activates or inhibits intracellular signaling pathways through G-protein signaling. 5-${\mathrm{HT}}_{1A}$ signaling is generally associated with anxiolytic effects, reducing anxiety levels [4]. In contrast, activation of 5-${\mathrm{HT}}_{2A}$ receptors by serotonin has been shown to exacerbate anxiety responses in mice [4]. Additionally, 5-${\mathrm{HT}}_{3}$, an ionotropic receptor, induces depolarization in serotonergic neurons and is also localized on neuronal membranes [5].

The serotonin transporter (SERT) regulates extracellular serotonin levels, and interestingly, altered SERT expression has been associated with anxiety across multiple species [6]. Selective serotonin reuptake inhibitors (SSRIs), a class of antidepressants, are designed to increase serotonin levels in the brain, a neurotransmitter critical for mood regulation and emotional well-being. These drugs have become essential in the treatment of various anxiety disorders triggered by stressful events.

In experimental studies, selective serotonin reuptake inhibitors, such as fluoxetine, are commonly used to treat anxiety disorders by inhibiting the reuptake of serotonin from synapses [7,8]. These drugs typically exhibit anxiogenic effects upon acute administration and anxiolytic effects with chronic administration in rodent models. SSRIs are considered first-line treatments for post-traumatic stress disorder (PTSD) [9,10,11,12,13,14,15], as anxiety symptoms in PTSD are often associated with reduced serotonin levels in limbic brain structures, such as the hippocampus [16,17,18,19,20,21]. The mechanisms underlying the paradoxical increase in anxiety during the initial phase of SSRI treatment are complex and not fully understood. However, several hypotheses offer insight into this phenomenon. In some cases, SSRI treatment disrupts the balance of monoamines in the brain, leading to an increase in serotonin levels. Prolonged SSRI treatment has been associated with heightened anxiety, potentially due to the desensitization of 5-${\mathrm{HT}}_{1A}$ autoreceptors, which regulate serotonin neurotransmission [22]. Recent findings have noted that the withdrawal of SSRIs after prolonged use is accompanied by a sharp increase in serotonin levels and heightened anxiety [21].

Stress-related anxiety disorders are not necessarily associated with reduced serotonin levels. In contrast, there is experimental evidence suggesting that increased serotonin secretion in the hippocampus is associated with heightened anxiety [23]. Serotonergic neurons projecting from the median raphe nucleus to the hippocampus, and from the dorsal raphe nucleus to the bed nucleus of the stria terminalis, are implicated in anxiety-like behaviors in rodents [24,25]. Notably, anxiety behaviors induced by chronic social defeat stress are associated with decreased expression of the SERT, MAO-A, and brain-derived neurotrophic factor (BDNF) genes in the raphe nucleus [26]. The mechanisms underlying serotonin-induced anxiety are linked to postsynaptic neuron receptors. For example, mice lacking the ionotropic 5-${\mathrm{HT}}_{3A}$ serotonin receptor exhibit anxiety-like behavior [27,28]. Additionally, the antagonism of the 5-${\mathrm{HT}}_{3A}$ receptor also induces anxiety-like behavior [29,30]. Anxiety-like behavior is also modulated by the interplay between 5-${\mathrm{HT}}_{3A}$ and 5-${\mathrm{HT}}_{2C}$ receptors [28]. 5-${\mathrm{HT}}_{2C}$ receptors in the bed nucleus of the stria terminalis (BNST) are involved in the development of anxiety [31].

Given the lack of consistent associations between stress-related anxiety disorders and reduced serotonin levels, there are risks associated with the effectiveness of SSRIs in these conditions. Moreover, the long-term use of SSRIs is associated with several adverse effects, such as impaired spermatogenesis, liver damage, and others [32,33]. To optimize the effects of SSRIs and minimize their side effects, a combined therapy with resveratrol (RES) has been proposed [33,34].

Resveratrol, a natural polyphenol derived from plants, is characterized by its ability to target multiple intracellular pathways, thereby exerting its cytoprotective effects [35,36,37,38]. RES may enhance the therapeutic efficacy of SSRIs while potentially mitigating their adverse effects [39]. Through the activation of sirtuins, followed by the subsequent activation of adenosine monophosphate-activated protein kinase (AMPK) [40,41], RES regulates the AMP/ATP ratio in cells, thus playing a critical role in the regulation of cellular energy metabolism [42]. In addition, RES acts as a free radical scavenger, providing a direct antioxidant effect [43]. The serotonin transporter, located on the presynaptic membrane of neurons, is also a target for RES. Consequently, RES and SSRIs may act synergistically [44]. Moreover, incorporating RES into the treatment protocol may enhance the anxiolytic effects of SSRIs due to its ability to target additional molecular pathways associated with anxiety beyond SERT. Notably, some of these targets may even be located outside the brain. In particular, RES downregulates the activity of liver enzyme 11β-hydroxysteroid dehydrogenase type 1 (11β-HSD-1), which is known to exacerbate stress-related anxiety by influencing blood glucocorticoid levels.

For over three decades, the predator stress model in rodents has been widely regarded as a model for PTSD [45,46]. However, during the induction of predator stress, the signal indicating the presence of a predator triggers a combination of anxiety and fear, which can potentiate the development of stress-related anxiety [47]. Recent studies have shown that, in conditions of chronic predator stress, the use of SSRIs alone is ineffective in correcting anxiety disorders [48]. It should be noted that this observation pertains only to the correction of behavioral disorders associated with stress, rather than PTSD, for which predator stress serves as a trigger.

The aim of this study was to evaluate the role of serotonergic signaling in the development of stress-related anxiety and to assess the effectiveness of new approaches for its treatment.

## 2. Materials and Methods

### 2.1. Chronic Predator Stress Paradigm

In this study, we employed a chronic predator stress (PS) paradigm. Previous research has utilized cat urine as a predator odor source for PTSD-related anxiety. In this protocol, anxiety-like behavior was observed only two weeks after the cessation of the PS paradigm, while an anxiolytic response predominated during the earlier stages. It has been demonstrated that replacing cat urine with fox urine, while maintaining similar timing and application quality, preserves stress-related anxiety [49]. This effect is partially linked to the enhanced activation of the amygdalar complex induced by fox urine [50].

### 2.2. Fox Urine Collection and Application

Urine was collected from sexually mature male domesticated silver-black foxes (*Vulpes vulpes*) during the autumn. Samples were obtained from several males, aliquoted, and stored at −18 °C for no more than one month. Using urine from mature males instead of females helps eliminate odor variations associated with the female reproductive cycle. The urine samples were thawed immediately before use. For stress exposure, 100 µL of urine was applied to a cotton pad, placed in a plastic Petri dish, and covered with nylon mesh to allow for the distribution of volatile compounds. The Petri dish was then placed in the animals’ home cages for 10 min daily, starting on day 5 of the experiment and continuing for 10 days.

### 2.3. Drug Administration

*Trans*-resveratrol (RES) was purchased from Sigma Aldrich Ltd. (St. Louis, MO, USA). RES was administered intraperitoneally, with daily injections from days 1 to 10 of the experiment. Solutions were prepared weekly and stored at room temperature. Both RES and the SSRIs, paroxetine (Paxil, JSC GlaxoSmithKline Trading, Moscow, Russia) and sertraline (Zoloft, Viatris, Freiburg, Germany), were dissolved in 99% dimethyl sulfoxide (DMSO) to ensure that the injection volume corresponded to 1 mL/kg of the animal’s body weight (100 mg/kg). We dissolved the chemicals in DMSO due to the good solubility of both resveratrol and SSRIs in this solvent. This approach allows for the administration of all drugs in a single injection.

### 2.4. Experimental Groups

**Figure 1:**
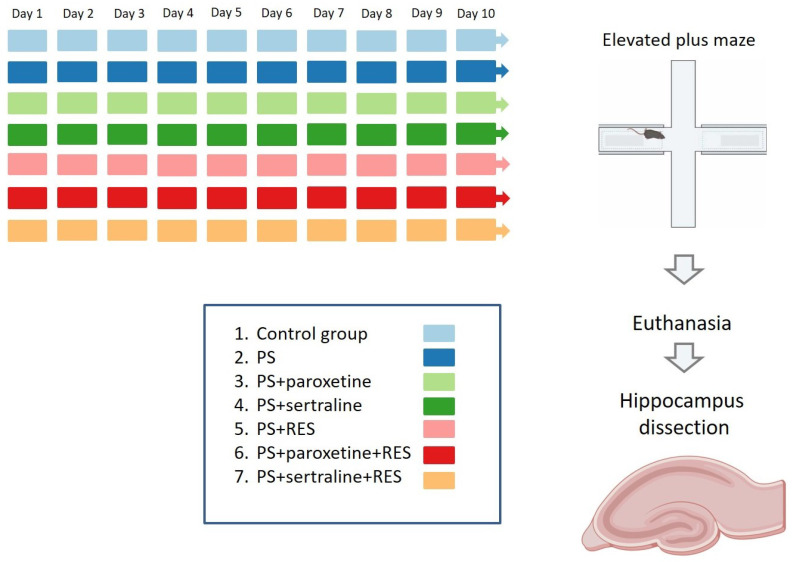
Timeline of predator stress exposures.

The animals were divided into the following groups (Figure 1):

1. Control: Rats treated with vehicle only for 10 days (*n* = 12);
2. PS: Rats exposed to chronic predator stress (*n* = 12);
3. PS + paroxetine: Rats administered an effective dose of paroxetine via intraperitoneal injection one hour before the onset of predator stress (*n* = 12);
4. PS + sertraline: Rats administered an effective dose of sertraline via intraperitoneal injection one hour before the onset of predator stress (*n* = 12);
5. PS + RES: Rats administered an effective dose of resveratrol via intraperitoneal injection one hour before the onset of predator stress (*n* = 12);
6. PS + paroxetine + RES: Rats administered effective doses of both paroxetine and resveratrol via intraperitoneal injection one hour before the onset of predator stress (*n* = 12);
7. PS + sertraline + RES: Rats administered effective doses of both sertraline and resveratrol via intraperitoneal injection one hour before the onset of predator stress (*n* = 12).

### 2.5. Behavioral Testing

The elevated plus maze (EPM) test was employed to measure anxiety-like behavior in rodents. This test can provide insights into conditions such as PTSD and other disorders characterized by anxious behavior. Additionally, it can serve as a component in the screening of novel compounds for their anxiolytic properties. The model is based on the aversion to open spaces, as indicated by the animal spending more time in the enclosed arms of the maze [51]. Anxiety levels in all rats were assessed using the EPM test, conducted with the standard EPM apparatus (TS0502-R3, OpenScience, Moscow, Russia). The test duration was set at 5 min. To minimize bias, control and experimental groups were tested simultaneously in a blinded manner, with the operator aware of the cage numbers but unaware of the group designations. Each rat was gently placed in the center of the maze, always facing the same open arm. The EPM test was conducted under low light conditions, specifically 30 lux of white light in the center of the maze. Each trial lasted 5 min, allowing the rat to explore the EPM freely. Between trials, the maze was thoroughly cleaned with a 30% ethanol solution and dried afterward. All experiments were conducted by the same experimenter, which has been shown to be critical for reducing data variability [52]. Behavioral recordings and tracking were carried out using the SMART video system, and data were analyzed with SMART 3.0 software. Key parameters measured included the number of entries into the open and closed arms, time spent in the open and closed arms, and freezing behavior.

### 2.6. Measurement of Hippocampal Concentrations of Monoamines and Its Metabolites

Hippocampal tissue was homogenized in 0.1 M perchloric acid and centrifuged at 7000× *g* for 15 min at 4 °C. The supernatants were filtered through a syringe equipped with a Whatman filter (0.2 µm pore size; MilliporeSigma, Burlington, VT, USA) and analyzed via high-performance liquid chromatography (HPLC). HPLC was conducted under isocratic conditions with electrochemical detection, utilizing a Hypersil BDS C18 reversed-phase column (250 × 4.6 mm, 5 µm; Thermo Fisher Scientific, Waltham, MA, USA). The mobile phase consisted of 75 mM phosphate buffer containing 2 mM citric acid, 0.1 mM octanesulfonic acid, and 15% (*v*/*v*) acetonitrile, adjusted to pH 4.6. Electrochemical detection was performed with a glassy carbon electrode at +780 mV. Monoamine and metabolite concentrations in tissue samples were quantified as pg/mg tissue, using an external calibration curve, as previously described [53]. The turnover of 5-HT was calculated as the ratio of [5-HIAA] to [5-HT], following the method of Slotkin et al. [54].

### 2.7. Measurement of mRNAs Concentrations

#### 2.7.1. RNA Isolation

Total RNA was isolated from hippocampal tissue using TRIzol Reagent (Invitrogen, Oxford, UK) according to the manufacturer’s protocol. RNA concentrations were quantified using a NanoDrop 2000 spectrophotometer (Thermo Fisher Scientific, Waltham, MA, USA). Sample purity was assessed by ensuring an optical density ratio (A260/A280) greater than 1.8. To further verify RNA integrity, the 18S/28S ribosomal RNA ratio was evaluated via electrophoresis on a 1.4% agarose gel.

#### 2.7.2. cDNA Synthesis and Real-Time PCR

Two µg of total RNA was used for complementary DNA (cDNA) synthesis using a high-capacity cDNA reverse transcription kit (Applied Biosystems, Thermo Fisher Scientific, Waltham, MA, USA). Quantitative real-time PCR was performed with the Evrogen 5 × qPCR mix-HS SYBR (Evrogen, Moscow, Russia). Primers were designed using Primer-BLAST software version Primer 3 Plus (National Center for Biotechnology Information, Boston, MA, USA). The specific primer sequences used in this study are listed in Table 1. Peptidyl prolyl isomerase A (PPIA) and glyceraldehyde 3-phosphate dehydrogenase (GAPDH) mRNA were used as internal controls.

### 2.8. Data Analyses

Data were analyzed using SPSS 24 (IBM, New York, NY, USA), STATISTICA 10.0 (StatSoft, Tulsa, OK, USA), RStudio 3.3.0+ (RStudio, Boston, MA, USA), and Excel v2308 (Microsoft, Redmond, WA, USA). The Shapiro–Wilk test was employed to assess the normality of data distribution. Results are presented as mean ± SEM or median (25th–75th percentile). Normally distributed data were analyzed using one-way ANOVA, followed by Tukey’s post hoc tests for group comparisons.

## 3. Results

### 3.1. Effects of Resveratrol and SSRIs on Anxiety-like Behavior in the Elevated Plus Maze Test

**Figure 2:**
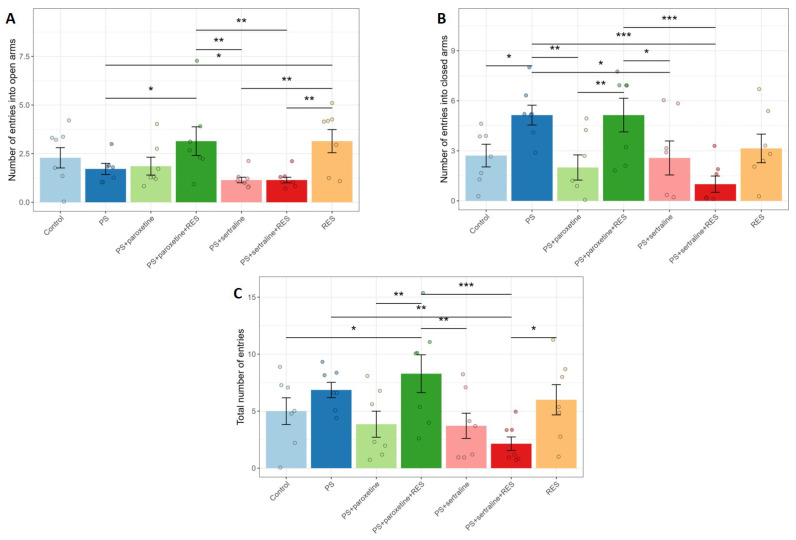
Effect of resveratrol and SSRI treatments on the number of entries into the open (**A**) and closed (**B**) arms of the EPM, and the total number of arm entries (**C**) in the elevated plus maze test in PS rats. * *p* < 0.05; ** *p* < 0.01; *** *p* < 0.001.

A one-way ANOVA revealed a significant impact of RES and SSRI treatment on the number of entries in the open (F(6,42) = 3.28, *p* = 0.009) and closed arms (F(6,42) = 3.79, *p* = 0.0041), as well as the time spent in the open (F(6,42) = 7.34, *p* < 0.0001) and closed arms (F(6,42) = 7.0, *p* < 0.0001) of the elevated plus maze (EPM) test (Figure 2). There was also a significant effect on the number of freezing episodes (F(6,42) = 4.29, *p* = 0.0011).

**Figure 3:**
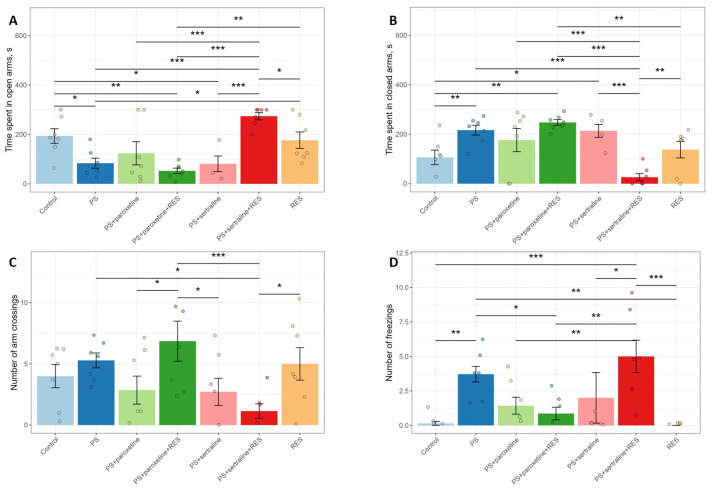
Impact of resveratrol and SSRI treatments on behavior in PS-subjected rats: (**A**) Time spent in the open arms of the EPM, (**B**) time spent in the closed arms, (**C**) number of arm crossings, and (**D**) number of freezing episodes. * *p* < 0.05; ** *p* < 0.01; *** *p* < 0.001.

Post hoc analysis showed that predator stress (PS) rats spent 100% more time in the closed arms (*p* = 0.0084) and 59% less time in the open arms (*p* = 0.0084) compared to the control group (Figure 3). The number of entries into the closed arms in PS rats was 89% higher (*p* = 0.0084), and the number of freezing episodes was 250% higher (*p* = 0.0004) compared to the control rats.

In the paroxetine-treated rats (“PS + paroxetine” group), the number of entries into the closed arms was reduced by 250% (*p* = 0.007), while in the sertraline-treated rats (“PS + sertraline” group), this parameter was decreased by 50% (*p* = 0.027) compared to PS rats. In RES-treated rats (“PS + RES” group), the time spent in the open arms was 229% higher (*p* = 0.01), and the time spent in the closed arms was 41% shorter (*p* = 0.025) compared to PS rats. Additionally, in the “PS + RES” group, the number of entries into the open arms was 184% higher (*p* = 0.034) than in the PS group, and there was a significant reduction in freezing episodes (*p* = 0.005).

Co-treatment with RES and paroxetine (“PS + paroxetine + RES” group) did not alleviate stress-related anxiety in PS rats. There were no significant differences between the PS group and the “PS + paroxetine + RES” group in the time spent in the open and closed arms. Moreover, in the “PS + paroxetine + RES” group, the time spent in the open arms was reduced by 350% (*p* = 0.001) compared to the “PS + RES” group. For the time spent in the closed arms, it was found to be 100% higher (*p* = 0.035) in the “PS + paroxetine + RES” group than in the “PS + RES” group. Additionally, in the “PS + paroxetine + RES” group, the number of entries into the open arms was 81% higher (*p* = 0.034), and freezing episodes were reduced by 71% (*p* = 0.025) compared to the PS group.

Co-treatment with RES and sertraline (“PS + sertraline + RES” group) was accompanied by an anxiolytic response in stressed rats. In the “PS + sertraline + RES” group, the time spent in the open arms was greater by 400% (*p* = 0.0015) than in the PS group and 137% higher (*p* = 0.044) compared to the “PS + RES” group. Correspondingly, in the “PS + sertraline + RES” group, the time spent in the closed arms was shorter by 92% (*p* = 0.003) than in the PS group and five times shorter (*p* = 0.021) than in the “PS + RES” group.

### 3.2. Impact of Resveratrol and SSRIs on Cathecholamines Metabolism in the Hippocampus of Predator-Stressed Rats

**Figure 4:**
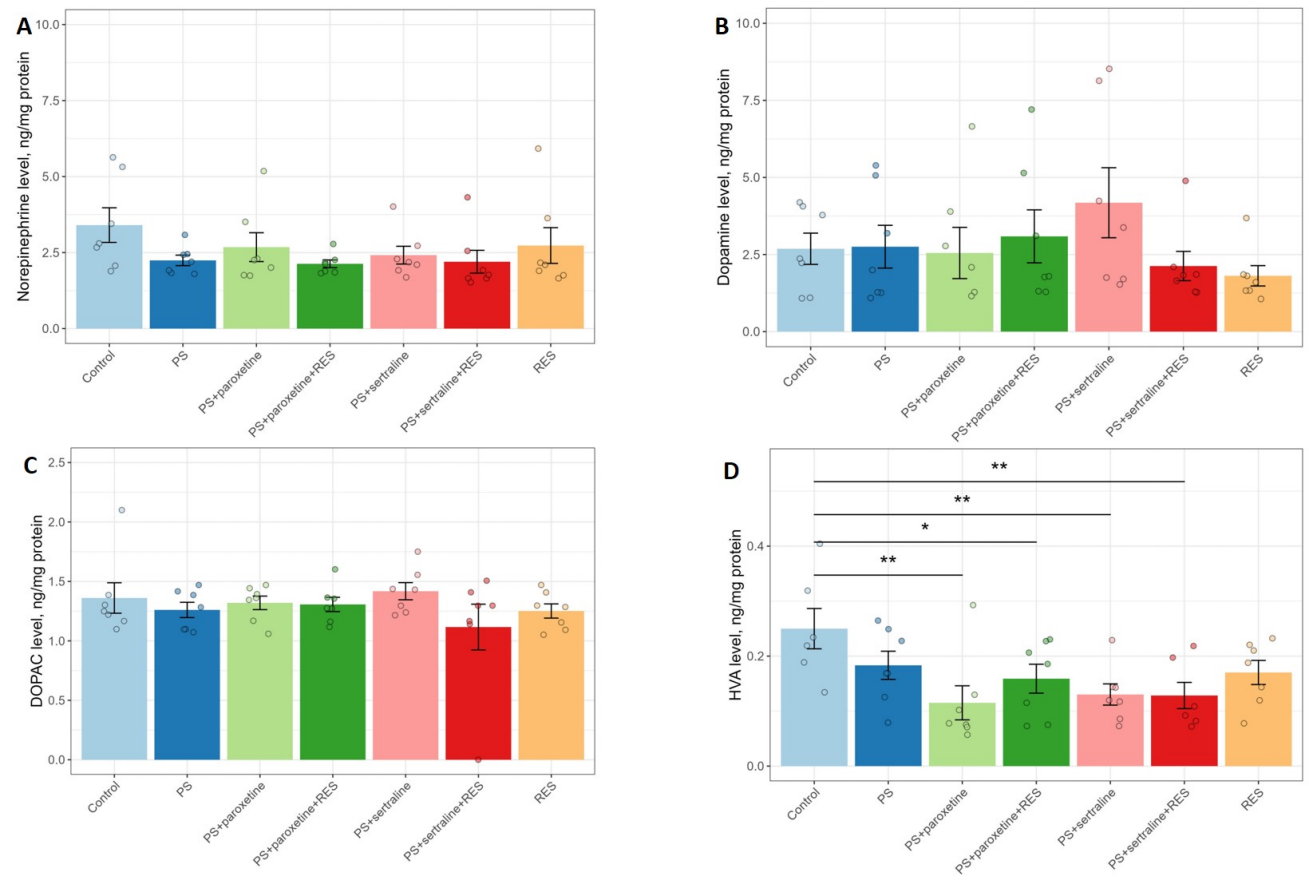
Impact of resveratrol and SSRI treatments on catecholamine metabolism in the hippocampus: (**A**) norepinephrine level, (**B**) dopamine level, (**C**) DOPAC level, and (**D**) HVA level. * *p* < 0.05; ** *p* < 0.01.

Figure 4 shows that neither RES nor SSRIs significantly affected catecholamine concentrations and metabolism in PS rats. There were no significant changes observed in noradrenaline (F(6,42) = 1.18; *p* = 0.33) and dopamine (*p* = 1.06; *p* = 0.39) concentrations, nor in DOPAC ((6,42) = 0.87; *p* = 0.53) and HVA ((6,42) = 0.84; *p* = 0.55) levels. However, significant differences were found in HVA concentrations ((6,40) = 2.65; *p* = 0.029), with higher levels observed in the control group compared to the “PS + paroxetine” (*p* = 0.0014), “PS + paroxetine + RES” (*p* = 0.026), “PS + sertraline” (*p* = 0.0041), and “PS + sertraline + RES” (*p* = 0.0049) groups.

### 3.3. Impact of Resveratrol and SSRIs on Serotonin Metabolism in the Hippocampus of Predator-Stressed Rats

**Figure 5:**
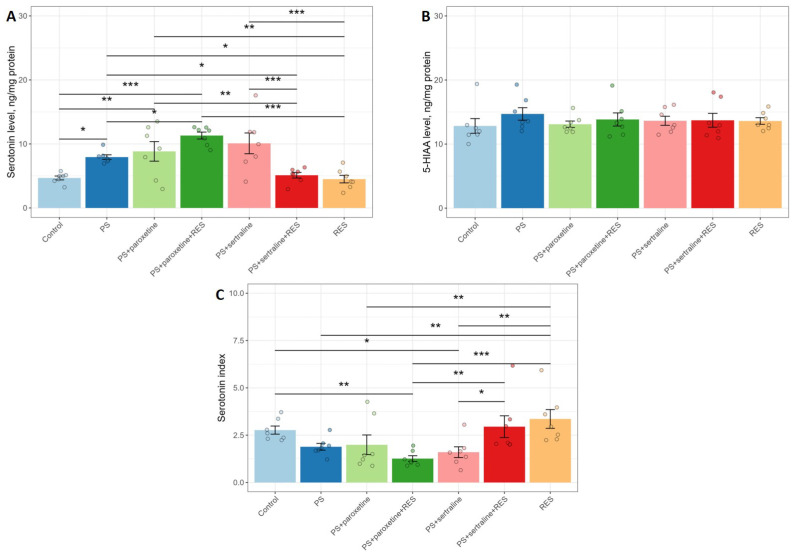
Impact of resveratrol and SSRI treatments on serotonin metabolism in the hippocampus: (**A**) serotonin level, (**B**) 5-HIAA level, and (**C**) serotonin index. * *p* < 0.05; ** *p* < 0.01; *** *p* < 0.001.

The data presented in Figure 5 suggest that both SSRI and RES treatments alter hippocampal 5-HT metabolism in PS rats. A one-way ANOVA revealed significant changes in 5-HT concentration (F(6,42) = 8.87, *p* = 0.0011). Although there were no significant changes in the concentration of 5-HIAA (F(6,42) = 0.36, *p* = 0.89), the 5-HT metabolic index (5-HIAA/5-HT) showed significant variation (F(6,42) = 4.51, *p* = 0.0011).

Post hoc analysis revealed that, in PS rats, the 5-HT concentration was 169% greater than in the control group (*p* = 0.024). Treatment with either paroxetine or sertraline did not significantly influence the elevated hippocampal 5-HT concentration in PS rats. In the “PS + paroxetine” group, the 5-HT concentration was 200% greater (*p* = 0.0004), while in the “PS + sertraline” group, it was 225% higher (*p* = 0.0002) compared to the control group.

RES treatment restored the 5-HT concentration to control levels. In the “PS + RES” group, the 5-HT concentration was 44% lower than in the “PS” group (*p* = 0.017). Co-treatment with RES and paroxetine exacerbated the increase in 5-HT concentration in PS rats. In the “PS + paroxetine + RES” group, the 5-HT concentration was 142% greater than in the “PS” group (*p* = 0.014), 241% higher than in the control group (*p* = 0.0001), and 251% higher than in the “PS + RES” group (*p* = 0.0001). In contrast, co-treatment with RES and sertraline restored 5-HT concentration in the hippocampus of PS rats. In the “PS + sertraline + RES” group, the 5-HT concentration was 36% lower than in the PS group (*p* = 0.038) and 42% lower than in the “PS + sertraline” group (*p* = 0.015). There were no significant differences between the “PS + sertraline + RES” group and the control group in hippocampal 5-HT concentration (*p* = 0.76).

In the “PS + RES” group, the value of the 5-HT metabolic index was 178% higher than in the “PS” group, whereas in the “PS + paroxetine + RES” group, the value of this index was 300% lower than in the “PS + RES” group (*p* = 0.008).

### 3.4. Effect of Resveratrol and SSRIs on Monoamine Oxidase and Catechol-O-Methyltransferase mRNA Expression in the Hippocampus of Predator-Stressed Rats

**Figure 6:**
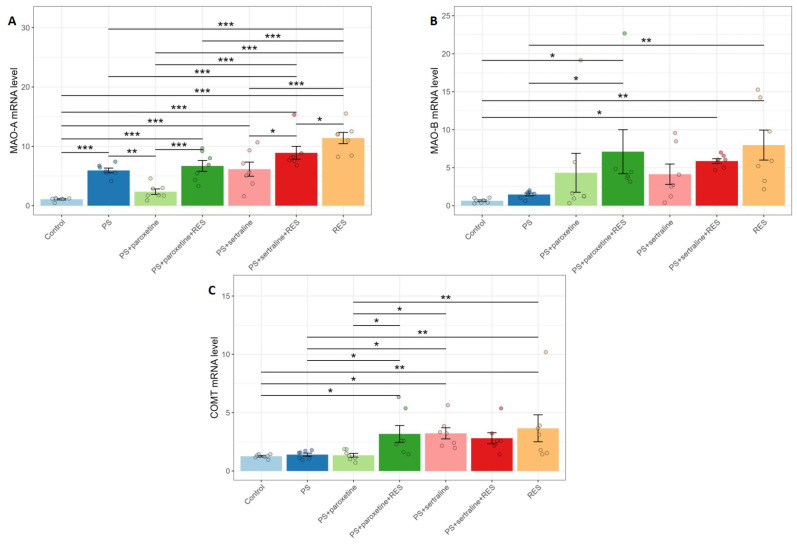
Impact of resveratrol and SSRI treatments on MAO-A (**A**), MAO-B (**B**), and COMT (**C**) mRNA levels in the hippocampus of PS-subjected rats. * *p* < 0.05; ** *p* < 0.01; *** *p* < 0.001.

One-way ANOVA analysis showed a significant influence of RES and SSRI treatments on MAO-A (F(6,42) = 18.57, *p* = 0.0001), MAO-B (F(6,41) = 2.58, *p* = 0.032), and COMT (F(6,42) = 3.25, *p* = 0.01)(F(6,42) = 3.25, *p* = 0.01) mRNA expression in the hippocampus of PS rats (Figure 6). In the “PS” group, MAO-A mRNA levels were sixfold higher than in the control rats (*p* = 0.0047). In the “PS + paroxetine” group, MAO-A mRNA levels were reduced to one-third of the levels observed in the “PS” group (*p* = 0.001). In contrast, sertraline treatment failed to reverse the elevated MAO-A mRNA levels. In the “PS + sertraline” group, the level of MAO-A mRNA was sixfold higher than in the control group (*p* = 0.0001).

RES treatment further increased the elevated MAO-A mRNA levels caused by PS exposure. In the “PS + RES” group, MAO-A mRNA levels were twofold higher than in the PS group (*p* = 0.0001). In the “PS + paroxetine + RES” group, the level of MAO-A mRNA was 44% lower than in the “PS + RES” group (*p* = 0.001) but remained 600% higher than in the control group (*p* = 0.0001). In the “PS + sertraline + RES” group, the level of MAO-A mRNA was 44% lower than in the “PS + RES” group (*p* = 0.0002), but still 600% greater than in the control group (*p* = 0.00001).

There were no significant differences between the control and “PS” groups in MAO-B mRNA levels (*p* = 0.76). However, in the “PS + RES” group, MAO-B mRNA levels were 545% higher than in the “PS” group (*p* = 0.003) and 1300% higher than in the control group (*p* = 0.00007). In both the “PS + sertraline + RES” (*p* = 0.13) and “PS + paroxetine + RES” (*p* = 0.11) groups, there were no significant differences compared to the “PS + RES” group.

Sertraline and RES treatments led to an increase in COMT mRNA levels in PS rats, whereas PS exposure alone did not affect COMT gene expression. In the “PS + RES” group, COMT mRNA levels were 257% higher than in the PS group (*p* = 0.0054). In the “PS + sertraline + RES” group, the MAO-A mRNA levels were 29% higher than in the “PS + sertraline” group (*p* = 0.038). Similarly, in the “PS + sertraline” group, COMT mRNA levels were 230% higher than in the PS group (*p* = 0.031). Co-treatment with RES and paroxetine also increased COMT gene expression in PS rats. In the “PS + paroxetine + RES” group, COMT mRNA levels were 225% higher than in the PS group (*p* = 0.036).

### 3.5. Effects of Resveratrol and SSRI Treatments on SERT and 5-${HT}_{3A}$ Receptor mRNA Expression in the Hippocampus of Predator-Stressed Rats

**Figure 7:**
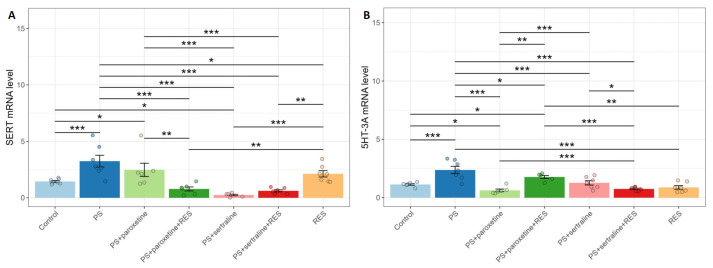
Impact of resveratrol and SSRI treatments on SERT (**A**) and 5-HT_3*A*_ (**B**) mRNA levels in the hippocampus of PS-subjected rats. * *p* < 0.05; ** *p* < 0.01; *** *p* < 0.001.

A one-way ANOVA analysis revealed a significant impact of stress exposure, as well as RES and SSRI treatments, on SERT (F(6,38) = 10.01, *p* = 0.0001) and 5-${\mathrm{HT}}_{3A}$ receptor expression in the hippocampus of PS rats (F(6,39) = 13.01, *p* = 0.0001) (Figure 7).

In PS rats, the levels of SERT mRNA were twofold higher than in the control group (*p* = 0.0042). Paroxetine treatment did not prevent the increase in SERT mRNA levels in stressed rats, with no significant difference between the PS and “PS + paroxetine” groups (*p* = 0.47). Conversely, sertraline treatment significantly suppressed SERT gene expression in PS rats. In the “PS + sertraline” group, SERT mRNA levels were 92% lower than in the PS group and 84% lower than in the control group (*p* = 0.023). RES treatment also prevented the elevation of SERT gene expression. In the “PS + RES” group, SERT mRNA levels were 35% lower than in the PS group (*p* = 0.039). Co-treatment with RES and either sertraline or paroxetine resulted in a greater reduction in SERT gene expression than RES treatment alone. In the “PS + sertraline + RES” group, SERT mRNA levels were 71% lower (*p* = 0.002), and in the “PS + paroxetine + RES” group, SERT mRNA levels were 63% lower (*p* = 0.008) compared to the “PS + RES” group.

Post hoc analysis revealed that, in PS rats, the levels of 5-${\mathrm{HT}}_{3A}$ mRNA were 235% higher than in control rats (*p* = 0.0001). Both paroxetine and sertraline treatments reduced 5-${\mathrm{HT}}_{3A}$ mRNA levels in PS rats. In the “PS + paroxetine” group, 5-${\mathrm{HT}}_{3A}$ mRNA levels were 53% lower than in the PS group (*p* = 0.0001) and 28% lower than in the control group (*p* = 0.046). In the “PS + sertraline” group, 5-${\mathrm{HT}}_{3A}$ mRNA levels were 53% lower than in the PS group (*p* = 0.0001), with no significant difference observed between the “PS + sertraline” and control groups (*p* = 0.56).

RES treatment also prevented the elevation of 5-${\mathrm{HT}}_{3A}$ mRNA levels in PS rats. In the “PS + RES” group, 5-${\mathrm{HT}}_{3A}$ mRNA levels were 73% lower than in the PS group (*p* = 0.0001). Co-treatment with RES and either paroxetine or sertraline effectively prevented the increase in 5-${\mathrm{HT}}_{3A}$ mRNA levels in PS rats. In the “PS + sertraline + RES” group, 5-${\mathrm{HT}}_{3A}$ mRNA levels were 71% lower (*p* = 0.0001), and in the “PS + paroxetine + RES” group, they were 26% lower (*p* = 0.026) than in the PS group. In the “PS + sertraline + RES” group, the 5-${\mathrm{HT}}_{3A}$ mRNA levels were 25% lower than in the “PS + sertraline” group (*p* = 0.038). Moreover, in the “PS + paroxetine + RES” group, the 5-${\mathrm{HT}}_{3A}$ mRNA levels were 31% higher than in the “PS + paroxetine” group (*p* = 0.038).

### 3.6. Influence of Resveratrol and SSRI Treatments on BDNF mRNA Expression in Predator-Stressed Rats

Regarding BDNF mRNA levels, one-way ANOVA analysis revealed a significant impact of SSRI and RES treatments on BDNF mRNA expression (F(6,42) = 4.17, *p* = 0.00025). Post hoc analysis showed that the PS paradigm did not affect BDNF mRNA levels (*p* = 0.27). Paroxetine treatment increased BDNF mRNA levels in PS rats. In the “PS + paroxetine” group, BDNF mRNA levels were twofold higher than in the PS group (*p* = 0.007). In contrast, sertraline treatment did not significantly affect BDNF mRNA levels in PS rats (*p* = 0.31).

**Figure 8:**
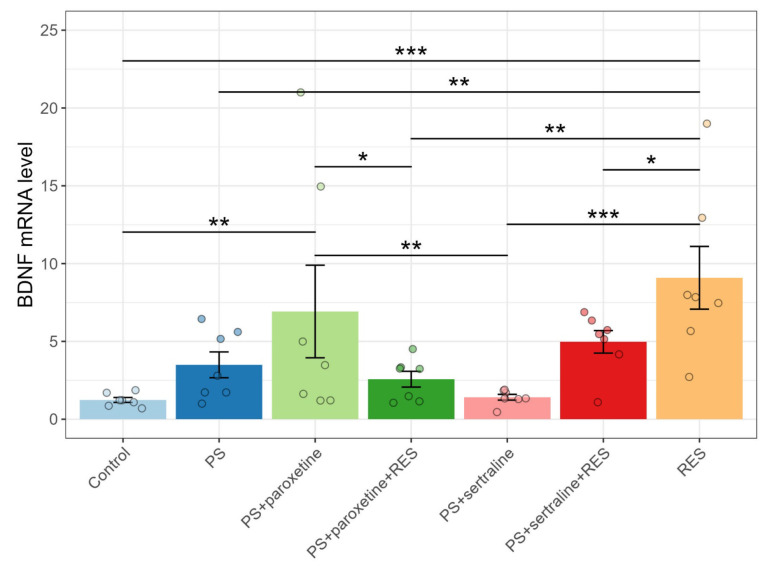
Impact of resveratrol and SSRI treatments on BDNF mRNA levels in the hippocampus of PS-subjected rats. * *p* < 0.05; ** *p* < 0.01; *** *p* < 0.001.

Data presented in Figure 8 show that RES treatment significantly elevated BDNF gene expression. In the “PS + RES” group, BDNF mRNA levels were 257% higher than in PS rats (*p* = 0.0003). Co-treatment with RES and SSRIs diminished the magnitude of this increase in BDNF mRNA levels in PS rats. In the “PS + paroxetine + RES” group, BDNF mRNA levels were 46% lower (*p* = 0.049), and in the “PS + sertraline + RES” group, BDNF mRNA levels were 72% lower (*p* = 0.0002) compared to the “PS + RES” group. In the “PS + sertraline + RES” group, the BDNF mRNA levels were 45% higher than in the “PS + sertraline” group (*p* = 0.038). In contrast, in the “PS + paroxetine + RES” group, the BDNF mRNA levels were 65% lower than in the “PS + paroxetine” group.

## 4. Discussion

In this study, the key findings are as follows: Chronic predator stress-induced anxiety is associated with an elevated concentration of serotonin in the hippocampus, along with simultaneous increases in SERT, 5-${\mathrm{HT}}_{3A}$, and MAO-A mRNA levels. SSRIs have failed to prevent or mitigate stress-related anxiety and did not significantly affect hippocampal serotonin levels in PS rats. In contrast, treatment with RES alleviates stress-related anxiety in these rats. The anti-anxiogenic effects of RES in PS rats are associated with a reduction in hippocampal serotonin levels, downregulation of SERT and 5-${\mathrm{HT}}_{3A}$ mRNAs, and upregulation of MAO-A and BDNF mRNAs. Moreover, co-treatment with RES and sertraline resulted in a more pronounced correction of stress-related anxiety compared to the effects of each drug administered separately.

PS can induce two types of anxiety: stress-related anxiety, which is directly related to stress, and PTSD-related anxiety, which arises from stress. Our objective was to determine whether these types of anxiety develop through different or similar mechanisms. Previously, we investigated the relationship between monoamine metabolism and gene expression in the hippocampus in the context of PTSD induced by predictive stress [55]. We found that PTSD-related anxiety is associated with decreased serotonin levels in the hippocampus. In that research, the analysis was conducted on the whole hippocampus. However, gene expression in the dorsal hippocampus correlates with cortical regions involved in information processing, while genes expressed in the ventral hippocampus are associated with emotion and stress [56]. Given this context, it would be rational to focus the analysis on the ventral hippocampus.

Nonetheless, it has been reported that the serotonergic system in the dorsal hippocampus is closely involved in the modulation of anxiety [57]. Therefore, we focused our analysis on the whole hippocampus, despite the functional differences between the dorsal and ventral regions. We examined monoamine metabolism and gene expression, viewing this approach as a miniature analysis of gene network abnormalities in stress-related anxiety. We have previously employed a similar methodology in PTSD analysis, considering the multitarget effects of RES as a result of its interference with gene expression. Anxiety disorders are conceptualized as failures in gene networks [58].

Resveratrol exerted a more pronounced correction of stress-related behavioral disorders compared to SSRIs. Treatment with resveratrol reduced stress-induced changes in the time spent in the arms and eliminated freezing behavior.

In stressed rats, SERT and 5-${\mathrm{HT}}_{3A}$ mRNA represent common molecular targets. Treatment with RES and SSRIs, either alone or in combination, reduced the expression of these genes. Co-treatment of RES with sertraline enhanced the anxiolytic effects of SSRIs. However, co-treatment with RES and paroxetine did not enhance the anxiolytic effects of SSRI. The PS paradigm did not affect catecholamine concentrations or their metabolites in the hippocampus. In contrast, the experimental PTSD model showed an increase in norepinephrine (NE) concentrations, accompanied by a decrease in dopamine (DA) and serotonin (5-HT) levels in the hippocampus, as well as changes in the concentrations of DA and 5-HT metabolites.

The essential distinction between the presented results and previous studies lies in the fact that this research does not specifically address PTSD. Instead, it focuses on stress-related anxiety. A comparison of neurochemical changes in the hippocampus suggests the presence of different molecular mechanisms underlying anxiety behavior of varying origins. Previously, researchers have reported the multiplicity of mechanisms involved in the development of anxiety behavior [59,60].

The increased expression of the 5-${\mathrm{HT}}_{3A}$ gene may be involved in the development of stress-related anxiety. This hypothesis is supported by the positive correlation between 5-${\mathrm{HT}}_{3A}$ mRNA levels and the time spent in closed arms (r = 0.78; *p* < 0.05), as well as the negative correlation with the time spent in open arms (r = −0.78; *p* < 0.05). The 5-${\mathrm{HT}}_{3A}$ receptor is localized in limbic and brainstem structures that regulate anxiety-related behavior and hypothalamic–pituitary–adrenal (HPA) axis activity [61].

As a ligand-gated ion channel, the 5-${\mathrm{HT}}_{3}$ receptor is expressed on interneurons throughout the brain [62]. Most species investigated to date show high levels of 5-${\mathrm{HT}}_{3}$ receptors in the amygdala and hippocampus compared to other forebrain regions [5]. Notably, 5-${\mathrm{HT}}_{3A}$-labeled glial processes are apposed to 5-${\mathrm{HT}}_{3A}$-immunoreactive axonal and dendritic profiles, some of which also express the SERT [63].

Adaptive changes in 5-${\mathrm{HT}}_{3}$ receptors have been shown to enable SERT knockout (SERT-KO) mice to survive despite inefficient serotonin inactivation [64]. Furthermore, increased 5-HT signaling at dorsal spine 5-${\mathrm{HT}}_{3}$ receptors has been implicated in visceral hypersensitivity observed in SERT-KO rats [65].

BDNF facilitates neuroplasticity in the hippocampus by attenuating signaling through the 5-${\mathrm{HT}}_{3}$ receptor. Thus, BDNF modulates hippocampal neuroplasticity through the serotonergic system [66]. It is proposed that both SERT and 5-${\mathrm{HT}}_{3}$ mRNAs may be simultaneously upregulated by corticosterone in stress paradigms [67,68]. Studies using 5-${\mathrm{HT}}_{3}$ knockout mice have demonstrated significant alterations in nociceptive processing and a reduction in anxiety-related behaviors, highlighting the receptor’s role in modulating both pain perception and anxiety [62].

The ionotropic 5-${\mathrm{HT}}_{3}$ receptor has emerged as a potential therapeutic target, as selective antagonists have been shown to reduce anxiety in rodents, primates, and humans [69]. Clinical studies indicate that antagonism of 5-HT type 3AB (5-${\mathrm{HT}}_{3AB}$) receptors in brain regions involved in mood regulation is an effective treatment for mood and anxiety disorders [70].

However, there is evidence that contradicts the understanding of the pro-anxiogenic effects of 5-${\mathrm{HT}}_{3A}$ receptors. In an animal model of PTSD, it has been shown that stimulation of 5-${\mathrm{HT}}_{3}$ receptors in the dorsal hippocampus prevents hippocampal autophagy and the development of PTSD-like behavior [71]. These findings further support the hypothesis that stress-related anxiety and PTSD-like anxiety are driven by distinct molecular mechanisms.

Increased hippocampal 5-HT concentration may be responsible for the elevated levels of SERT mRNA observed. SERT regulates 5-HT levels by reuptaking this monoamine from the synapse into the presynaptic neuron, where it is subsequently metabolized by monoamine oxidase (MAO-A). Notably, in PS rats, there is also an increase in MAO-A mRNA levels in the hippocampus.

The anti-anxiogenic effects of RES treatment are associated with a reduction in hippocampal 5-HT concentration and a concurrent decrease in SERT mRNA levels. Additionally, RES treatment in PS rats is linked to a decrease in 5-${\mathrm{HT}}_{3A}$ mRNA levels. Thus, RES appears to target the expression of both SERT and 5-${\mathrm{HT}}_{3A}$ genes in the hippocampus of PS rats. Furthermore, RES treatment upregulates the expression of MAO-A, MAO-B, and BDNF genes.

It is plausible that RES increases mRNA levels through a sirtuine 1 (SIRT1)-dependent pathway [72]. SIRT1 influences mood by deacetylating NHLH2, which in turn activates MAO-A transcription. In SIRT1-overexpressing mice, MAO-A protein levels are elevated, and SIRT1’s activity modulates MAO-A through NHLH2. Typically, activation of the SIRT1/NHLH2/MAO-A pathway is associated with increased anxiety behavior. However, in the PS paradigm, RES treatment was observed to decrease brain MAO-A activity [73].

Moreover, tissue inhibitors of MAO, known as tribulins, can reduce enzymatic activity even when MAO-A gene expression is increased [74]. It is conceivable that the low MAO activity following RES treatment could trigger an enhancement of MAO-A gene expression to manage the elevated 5-HT levels in the hippocampus of PS rats. RES has been demonstrated to alleviate anxious behavior by upregulating BDNF levels in the hippocampus. This upregulation is associated with an increase in synaptosome proteins, which support neuroplasticity, such as synaptic RAS GTPase activation protein (SynGAP), postsynaptic density protein 95 (PSD95), synapsin-1, and synaptogmin-1. This process is dependent on SIRT1 [75,76].

The ability of RES to enhance synaptic plasticity is particularly advantageous for addressing stress-related anxiety disorders, as synaptic stability significantly impacts neurotransmitter efficacy [77]. Overall, the neuroprotective properties of RES contribute to its effectiveness in treating anxiety disorders by improving synaptic plasticity and neurotransmitter function.

SSRI treatment also affects 5-${\mathrm{HT}}_{3A}$ gene expression in PS rats. Both paroxetine and sertraline significantly reduced 5-${\mathrm{HT}}_{3A}$ mRNA levels, with a more pronounced effect compared to RES. Notably, sertraline led to the greatest reduction in SERT mRNA levels, whereas paroxetine did not impact SERT gene expression in the hippocampus of PS rats.

In contrast to RES, neither sertraline nor paroxetine was able to normalize hippocampal 5-HT levels in PS rats. In paroxetine-treated rats, the elevated 5-HT levels were associated with a reduction in MAO-A gene expression in the hippocampus. Interestingly, while sertraline treatment resulted in a reduction in BDNF gene expression, there was still some alleviation of anxiety behavior. Conversely, paroxetine-treated rats exhibited a noticeable increase in BDNF gene expression but failed to show improvement in anxiety-related behavior.

This paradox suggests that future research should focus on downstream signaling pathways, particularly the BDNF-TrκB pathway, to better understand the mechanisms underlying these differential effects.

Co-treatment with RES and paroxetine exacerbated the 5-HT overload in the hippocampus of PS rats, while simultaneously causing a marked reduction in SERT gene expression. The observed increase in COMT gene expression in paroxetine-treated rats suggests that this postsynaptic enzyme plays a role in the regulation of catecholamine metabolism under paroxetine treatment.

In contrast, co-treatment with RES and sertraline led to a more significant improvement in anxiety-like behavior than RES treatment alone. Notably, this co-treatment restored serotonin levels to control values, whereas co-treatment with paroxetine failed to alleviate the serotonergic overload in PS rats. The normalization of serotonin in rats co-treated with RES and sertraline levels was associated with the overexpression of the MAO-A gene, an enzyme responsible for the oxidative deamination of this monoamine. In contrast, in rats co-treated with RES and paroxetine, the elevation of MAO-A mRNA levels was less pronounced compared to their counterparts receiving sertraline. Furthermore, co-treatment with sertraline resulted in a more significant increase in BDNF mRNA levels than that observed with paroxetine in PS rats administered RES. Additionally, the combination of RES and paroxetine suppressed the expression of SERT and 5-${\mathrm{HT}}_{3A}$ genes but did not lead to an improvement in anxiety-like behavior, unlike the RES and sertraline combination. These findings suggest that these genes likely form part of an overexpression of the BDNF gene and may further contribute to the alleviation of the coordinated gene network within serotonergic neurons, where their expression changes in response to fluctuations in serotonin levels. Paradoxically, paroxetine-treated rats exhibited a notable increase in BDNF gene expression but did not show any improvement in anxiety-related behavior. In contrast, RES has been demonstrated to alleviate anxious behavior by upregulating BDNF mRNA levels in the hippocampus. Notably, the increase in BDNF levels in PS rats treated with RES is quantitatively much greater than that observed in PS rats treated with paroxetine. Therefore, it would be beneficial for future research to establish a cut-off value for BDNF mRNA expression, similar to what has been done in previous PTSD studies for anxiety indices. Determining whether BDNF levels are sufficient to elicit a biological effect in specific contexts would be an intriguing area of investigation. However, this labor-intensive task warrants a separate study.

The obtained data suggest that RES at a dose of 100 mg/kg effectively improves anxiety-related behavior and may rival the efficacy of SSRIs. Interestingly, in the case of stress-related anxiety, an increase in hippocampal 5-HT concentration was observed, whereas in PTSD models, a decrease in hippocampal 5-HT levels was noted.

**Figure 9:**
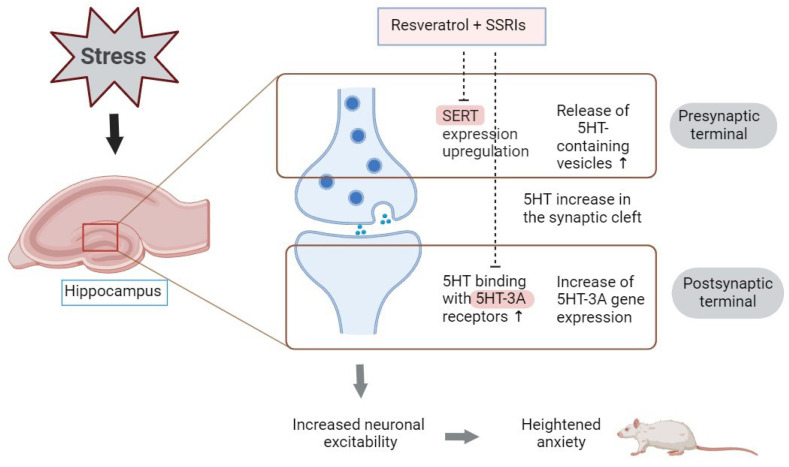
Proposed serotonergic mechanism of stress-related anxiety.

In Figure 9, the proposed mechanism for the development of stress-related anxiety is presented. A key component in the pathogenesis of anxiety is the hyperexpression of 5-${\mathrm{HT}}_{3A}$ receptors. As this receptor is ionotropic, it mediates the depolarization and excitation of serotonergic neurons. Additionally, under stress, this type of receptor downregulates the HPA axis, further exacerbating anxiety responses [78].

At first glance, some of the data presented here may not seem to support the proposed serotonergic mechanism of anxiety. For example, co-treatment with RES and paroxetine suppressed SERT and 5-${\mathrm{HT}}_{3A}$ expression but did not improve anxiety-like behavior, in contrast to co-treatment with RES and sertraline. To resolve this, it may be useful to focus on the quantitative aspects of these changes. In RES + sertraline-treated rats, hippocampal 5-${\mathrm{HT}}_{3A}$ mRNA levels were 45% lower than in those treated with RES + paroxetine. Therefore, it would be useful to define a cut-off value for 5-${\mathrm{HT}}_{3A}$ mRNA expression, as this could provide a clear understanding of whether its reduction is sufficient to achieve the pharmacological effects of RES and SSRIs. It is possible that the combination of sertraline and RES reduces anxiety by lowering 5-${\mathrm{HT}}_{3A}$ mRNA levels below this cut-off, whereas the combination of paroxetine and RES does not. Nevertheless, it is important to note that the use of the cut-off criterion in relation to 5-${\mathrm{HT}}_{3A}$ receptors can only rationally explain some of the pharmacological effects of RES and SSRIs discussed here. Specifically, there are challenges in providing a consistent explanation for certain contradictions. For instance, paroxetine significantly reduced the 5-${\mathrm{HT}}_{3A}$ mRNA levels but did not alleviate anxiety-like behaviors. However, it is worth clarifying that, while paroxetine treatment did not improve anxiety levels, it did mitigate other forms of stress-induced behavioral disturbances. This was evident by a reduction in freezing behavior, an indicator of fear, in rats treated with paroxetine. In stressed rats, increased anxiety levels were paralleled by elevated freezing behavior. Moreover, a significant positive correlation (r = 0.69, *p* < 0.05) was found between freezing behavior and 5-${\mathrm{HT}}_{3A}$ mRNA levels. Indeed, paroxetine treatment did not improve anxiety-like behavior despite low 5-${\mathrm{HT}}_{3A}$ mRNA levels. However, unlike treatment with RES, or combined RES and sertraline therapy, paroxetine—whether administered alone or in combination with RES—did not reduce serotonin levels in the hippocampus. Interestingly, there is a positive correlation (r = 0.72, *p* < 0.05) between serotonin concentration and the time spent in the closed arms of the elevated plus maze. It is also noteworthy that, in paroxetine-treated rats, the elevated serotonin levels were associated with a decreased expression of MAO-A mRNA.

Therefore, to fully assess the pharmacological effects of RES and SSRIs, it would be useful to establish a cut-off not only for 5-${\mathrm{HT}}_{3A}$ mRNA but also for other hippocampal markers sensitive to drug action. However, it is clear that the cut-off approach serves as a helpful clarification for interpreting results, yet it remains insufficient for fully describing the serotonergic mechanism of anxiety. It is imperative for the gene expression and monoamine levels in other brain regions, such as the median raphe nucleus, dorsal raphe nucleus, bed nucleus of the stria terminalis, and periaqueductal gray, to be investigated to better understand the anxiolytic mechanisms of RES and SSRIs. Determining cut-offs in these regions may also prove beneficial. Thus, the identification of cut-offs and the analysis of the serotonergic system in other brain regions are not alternatives, but rather complementary approaches. Although these apparent paradoxes can be explained based on the obtained results, future research should also include analyses of other brain regions to further elucidate the mechanisms at play.

It is noteworthy that the PS paradigm used in this study is characterized by decreased plasma corticosterone (CORT) concentration and increased hepatic 11β-hydroxysteroid dehydrogenase type 1 activity [49]. Interestingly, the increase in 11β-HSD-1 activity has been associated with heightened stress-related anxiety, suggesting its potential role in the exacerbation of anxiety responses despite the reduced CORT levels.

Moreover, in another version of the PS paradigm, reduced glucocorticoid levels and increased excitability of serotonergic neurons are closely correlated. When anxiety behavior arises through this mechanism, SSRIs, which primarily function by increasing 5-HT concentrations in synapses, encounter significant challenges in exerting their anxiolytic effects [79]. The increased excitability of serotonergic neurons, combined with lower glucocorticoid levels, may introduce complexities in the regulation of serotonin signaling, potentially diminishing the therapeutic efficacy of SSRIs in stress-related anxiety.

However, co-treatment with RES significantly alleviated this issue. As a multi-target compound, RES has the capacity to address several key pathogenetic elements of anxiety, including disturbances in neurocircuitry, neurotransmitter imbalances, oxidative stress, mitochondrial dysfunction, and loss of neuroplasticity. By modulating multiple pathways, RES helps restore overall neuronal function and reduces anxiety more effectively than therapies that target only serotonin reuptake, such as SSRIs [80].

Meanwhile, resveratrol is metabolized at a high rate, which limits its potential as a therapeutic drug. There are several approaches to stabilizing resveratrol: (A) the synthesis of its stable derivatives, (B) the development of nanocapsules, and (C) co-administration with other drugs [81]. It is likely that the co-administration of resveratrol with sertraline, but not with paroxetine, could have successfully addressed this issue. Future research plans include further exploration of this approach, testing different doses of resveratrol in combination with other SSRIs to enhance its therapeutic potential.

The main limitations of this study arise from its preliminary nature. The research was conducted exclusively in the hippocampus, despite the fact that serotonergic neurons originating from the raphe nuclei project to other critical brain regions, including the prefrontal cortex, amygdala, hypothalamus, and striatum. Future studies should unquestionably extend the analysis to these areas. Additionally, this study does not address the expression of other serotonin receptors, which are known to modulate serotonin signaling. Another limitation is the lack of protein expression data to complement the gene expression findings. These limitations will be addressed in future studies, which will aim to provide a more comprehensive understanding of the serotonergic mechanisms underlying anxiety in chronic stress.

## 5. Conclusions

This pilot study identifies novel molecular targets underlying the anti-anxiogenic effects of resveratrol. Future research should explore the specific contribution of 5-${\mathrm{HT}}_{3A}$ receptors to the pathophysiology of stress-induced anxiety. Additionally, investigating the role of other serotonin receptor subtypes in serotonin regulation is crucial. The potential interactions between serotonin receptors and other receptor systems, such as dopaminergic receptors, warrant further investigation. An in silico assessment of resveratrol’s binding affinity to serotonin receptors should also be considered. Finally, evaluating the anxiolytic efficacy of resveratrol in combination with other established anxiolytics remains an important avenue for future research.

## Figures and Tables

**Table 1:** The primer sequences used for the evaluation of mRNA expression levels.

Primer’s Name	Primer’s Sequence (5′ ⟶ 3′)	Temperature (°C)
5-HT3	**F** CTGTCCTCCATCCGCCACTCC **R** CAGCAGCCTGTCCAGCACATATC	60
SERT	**F** ATAGCCAACATGCCAGCATCCAC **R** ACCACGATGAGCACGAACCATTC	68
MAO-A	**F** GCCAGGAACGGAAATTTGTA **R** TCTCAGGTGGAAGCTCTGGT	64
MAO-B	**F** TGGGAAGATTCCAGAGGATG **R** GCTGACAAGATGGTGGTCAA	60
BDNF	**F** GAAAGTCCCGGTATCAAAAG **R** CGCCAGCCAATTCTCTTTTTG	60
COMT-105	**F** CTGGAGAAATGTGGCCTGCT **R** GCTGCTGCTCCCTCTCACAT	60

## Data Availability

The original contributions presented in the study are included in the article, further inquiries can be directed to the corresponding authors.
